# Rethinking bias and truth in evidence‐based health care

**DOI:** 10.1111/jep.13010

**Published:** 2018-08-06

**Authors:** Sietse Wieringa, Eivind Engebretsen, Kristin Heggen, Trish Greenhalgh

**Affiliations:** ^1^ Institute of Health and Society University of Oslo Oslo Norway; ^2^ Department of Continuing Education/EBHC University of Oxford Oxford UK; ^3^ Primary Care Health Sciences Oxford University of Oxford Oxford UK

**Keywords:** epistemology, evidence‐based medicine, practical reasoning, science

## Abstract

In modern philosophy, the concept of truth has been problematized from different angles, yet in evidence‐based health care (EBHC), it continues to operate hidden and almost undisputed through the linked concept of “bias.” To prevent unwarranted relativism and make better inferences in clinical practice, clinicians may benefit from a closer analysis of existing assumptions about truth, validity, and reality.

In this paper, we give a brief overview of several important theories of truth, notably the ideal limit theorem (which assumes an ultimate and absolute truth towards which scientific inquiry progresses), the dominant way truth is conceptualized in the discourse and practice of EBHC.

We draw on Belgian philosopher Isabelle Stengers' work to demonstrate that bias means one thing if one assumes a world of hard facts “out there,” waiting to be collected. It means something different if one takes a critical view of the knowledge‐power complex in research trials. Bias appears to have both an unproductive aspect and a productive aspect as argued by Stengers and others: Facts are not absolute but result from an interest, or interesse: a bias towards a certain line of questioning that cannot be eliminated.

The duality that Stengers' view invokes draws attention to and challenges the assumptions underlying the ideal limit theory of truth in several ways. Most importantly, it casts doubt on the ideal limit theory as it applies to the single case scenario of the clinical encounter, the cornerstone of EBHC. To the extent that the goal of EBHC is to support inferencing in the clinical encounter, then the ideal limit as the sole concept of truth appears to be conceptually insufficient. We contend that EBHC could usefully incorporate a more pluralist understanding of truth and bias and provide an example how this would work out in a clinical scenario.

## 1 INTRODUCTION

Truth is and has been one of the most central and extensively debated topics in philosophy.^1^ Whilst philosophers are keen to understand the nature of truths and what makes them true, scholars of evidence‐based health care (EHBC) however have tended to more interested in bias. A simple word search on PubMed gives about 181 000 hits for bias and just 22 000 hits for truth. Epidemiologists generally address the topic of bias by describing categories of kinds of bias and offering suggestions on how to minimize or eliminate each.^2^, ^3^, ^4^, ^5^, ^6^, ^7^, ^8^, ^9^

The focus on research methodology (specifically the practicalities of experimentation) and clinical application (specifically poor reasoning in the clinical context) may go some way to explaining why bias became so important in evidence‐based practice and hence why truth as a concept was largely neglected. As a result, however, the dominant discourse of bias in EBHC is inclined to wrongly normalize truth as a given. It overlooks that the debate around truth is far from resolved and indeed remains a live topic in philosophy and the humanities.

Also, the way bias is understood in EBHC does not seem to provide sufficient argumentation against alternative facts, factoids, and post truths. Renewed concern of falsities being propagated on an unprecedented scale through social media has inspired many from outside^10^, ^11^, ^12^ and within the research community^13^, ^14^ to come up with new solutions to counter those, but these arguments tend not to address the more fundamental question: how do truths differ from untruths (and what is the nature of the grey zone in the middle)?

Most importantly, the discourse on bias in EBHC assumes that truth is unproblematic and that, therefore, the right decisions will emerge once all sources of biases are defeated. This flawed assumption constrains thinking of what good decision‐making in the clinical encounter actually entails.

Belgian philosopher Isabelle Stengers offers an alternative view in her books *Sciences et Pouvoirs* 15 and *Power and Invention*.^16^ She sees bias as productive and necessary to forge facts. Drawing on her work and that of others, we set out in this paper to uncover some of the fundamental beliefs and assumptions on bias and truth that drive and constrain what we can know and do in clinical practice. We conclude that much could be achieved by turning our attention to discussing, teaching, and extending theories of truth and considering its relationship to bias in evidence‐based practice.

## 2 THE IDEAL LIMIT THEOREM OF TRUTH

The philosophical debate on truth spans several millennia. However, contemporary authors on the subject usually address a set of dominant views in one way or the other.^17^, ^18^, ^19^, ^20^ Correspondence theories of truth hold that what is true should somehow represent how reality actually is. Coherence theories see truth as what coheres with a whole set of beliefs. Pragmatist theories of truth refer to what works in practice. Constructivist theories of truth are concerned with how scientists interpret the world and how particular interpretations come to shape research traditions and empirical choices.^21^ Deflationist theories attribute limited significance to the concept of truth and question what it actually means to say something is true. These broad philosophical positions are by no means exhaustive; many other views on truth exist in the literature.

We suggest (in lack of empirical data) that the epidemiological perspective taken by most EBHC scholars implicitly is a concept of truth known as the ideal limit theorem^17^, ^22^ as commonly attributed to late 19th century American philosopher C.S. Peirce.^23^ Although Peirce propagated several concepts of truth,^24^ he is most famous for the belief that the truth would reveal itself in the long run, over many events in many communities now and in the future. He states:

Truth is that concordance of an abstract statement with the ideal limit towards which endless investigation would tend to bring scientific belief, which concordance the abstract statement may possess by virtue of the confession of its inaccuracy and one‐sidedness, and this confession is an essential ingredient of truth^25^

This idea of the long run finds its way to health care research via Fisher's publications in the 1920s on scientific experiments,^26^ and Bradford Hill's work on clinical trials in the 1930s^27^ that led to the important methodological development of the randomized controlled trial (RCT) in which participants were allocated to treatment and nontreatment groups with the explicit goal of eliminating bias.^28^ In The Lancet in 1937, Bradford Hill writes:

The reason why in experiments in the treatment of disease the allocation of alternate cases to the treated and untreated groups is often satisfactory, is because no conscious or unconscious bias can enter in, as it may in any selection of cases, and because in the long run [italics in original] we can fairly rely upon this random allotment of the patients to equalise in the two groups the distribution of other characteristics that may be important. Between the individuals within each group there will often be wide differences in characteristics, for instance in body‐weight and state of health, but with large numbers we can be reasonably sure that the numbers of each type will be equally, or nearly equally, represented in both groups.^27^

Further promotion of the RCT by Cochrane^29^ and others in the 1970s laid the cornerstones of the EBHC movement.^30^ The early evidence‐based medicine (EBM) protagonists developed a hierarchy (or pyramid) of evidence with systematic reviews on top and case studies at the bottom,^31^, ^32^ on the basis that methodological designs at the top of this hierarchy were less prone to bias (and therefore more likely to lead scientists to the truth).

Viewed from the ideal limit concept, bias is depicted negatively as something that distorts the truth (which would otherwise be pure, universal, and attainable). Bias has been defined by EBHC scholars as “a cause of systematic error,”^5^ a “deviation in judgement,”^33^ an inability “to approximate the truth,”^4^ “a deviation from the truth,”^3^ and “the lack of internal validity or incorrect assessment of the association between an exposure and an effect in the target population in which the statistic estimated has an expectation that does not equal the true value.”^2^

As EBHC deliberately sets out to support clinical decision‐making in the single case scenario of the clinical encounter,^34^ *cognitive biases* refer to failures in clinical reasoning to correctly estimate the ideal limit (“true” prevalence, incidence, and risk), inspired partly by the works of Kahneman and Tversky.^33^, ^35^, ^36^, ^37^ These authors explored “the cognitive and the psychophysical determinants of choice in risky and riskless contexts”^38^ through typically well‐controlled studies using economic modelling and gambles. These notions were further developed by others for health care and include, for example, anchoring bias (undue emphasis given to a salient feature in the consultation), ascertainment bias (thinking shaped by prior expectation), search satisficing (having found one diagnosis, others are neglected), and many more.^33^

The various markers of methodological quality proposed by EBHC scholars—for example, to use the RCT design where appropriate and possible, to ensure adequate sampling and sample size, to analyse data on an intention to treat basis, and to publish all findings whether positive, negative, or equivocal^39^—are all ultimately designed to reduce bias and hence help scientists get ever closer to the ideal limits as the facts “out there.”

## 3 BIAS AS INTERESSE

But bias can be understood very differently from a knowledge‐power structure view as developed by Isabelle Stengers. She refers to interest in the original Latin sense: “interesse” meaning “to be situated between.”

She claims that no scientific proposition can, in any relevant sense, be called “true” if it has not attracted “interests.” Knowledge is, according to her, built on attachments, interests, and attractions. In *Power and Inventions*, she writes:

However, in most cases, a “fact” is not in and of itself so talkative. Its significance, as well as its recognition, involve a history that is produced by active strategies. Whom will it “interest” – that is, who will agree to associate his [sic] research program with it, to be situated by it, that is, let it “be between” (inter‐esse) his own questions and those that produced it? This is a crucial question, because what we have to call the creation of a reality depends on it. Indeed, reality is of course not what exists independently of human beings, but that which demonstrates its existence by bringing together a multiplicity of disparate interests and practices.^16^

In *Sciences et Pouvoirs*, she states in her introduction (Au nom de la science) that she intends to refute the traditional claim that science and interests are opposites and that science (and associated claims of objectivity and truth) can be obtained only by “purifying” science from subjective interests. On the contrary, facts become facts by attracting subjective interests:

They deserve this title [of being a fact], not because their existence has been proven by empirical science (since subsequent empirical studies can always destroy it using new technical approaches and sources); but because they have become a crossroads for heterogeneous practices, each with different interests, each of which has required the phenomena in question to be able to relate reliably to their questions and interests.^15^ (Quote translated from French by authors)

Stengers' argument should not be mistaken for a social constructivist claim. She does not argue that facts are mere social constructs. She does not claim that all propositions about reality are equally true and that their status as facts (or nonfacts) depends solely on social consensus. Rather, she claims that truths are accepted as facts only when they become interesting. When Pasteur's discovery of lactic acid fermentation became a major scientific breakthrough, it was not mainly because of the discovery itself or the rigour of his methods but because he was “able to make the social powers work in his favour.”^15^

Of course, Pasteur's genius was to link the question of microorganisms to questions of interest to industrialists, farmers and doctors. Why does beer become bad? Why do our cows die of anthrax? How to fight against epidemics?^15^ (Quote translated from French by authors)

She goes on to argue that there are many truths that never become accepted as facts:

It is impossible to count the number of proposals that “could have” had a chance and become viable, and were rejected with a shrug ... because for a laboratory “result” to be the origin of a process, a device, a product of interest to the life of society, it is necessary that it has changed hands, that it has become “interesting” for a collection of actors other than the “competent colleagues.”^15^ (Quote translated from French by authors)

Her argument is that since facts are in the most fundamental sense built on interests, all science is (in this sense) inherently biased. Facts are never absolute but result from an interest, a bias towards a certain line of questioning.

This perspective aligns broadly with that of other feminist philosophers, notably Martha Nussbaum, who have argued that facts are invariably value‐laden and, furthermore, that an actor's emotional and moral position in relation to a “fact” (reflexively surfaced and examined) strengthens rather than weakens scholarly inquiry.^40^

Stengers' perspective also has some resonance with Foucault's important argument that power and knowledge are intimately related. The quest for knowledge makes people “visible” by subjecting them to observation, quantification, and classification. Moreover, this visibility is integrated into people's own vision of themselves: “He who is subjected to a field of visibility, and knows it, assumes responsibility for the constraints of power; he makes them play spontaneously upon himself.”^41^ ^(p202)^ Knowledge thus enables governance by governing people's vision and reasoning. This also implies that without discipline and without controlling people's gaze, there would be no knowledge. In fact, Foucault insists on the productive role of power: “We must cease once and for all to describe the effects of power in negative terms: it “excludes,” it “represses,” it “censors,” it “abstracts,” it “masks,” it “conceals.” In fact, power produces; it produces reality; it produces domains of objects and rituals of truth. The individual and the knowledge that may be gained of him belong to this production.”^41^ ^(p194)^

## 4 DUALITY OF BIAS CHALLENGING THE IDEAL LIMIT

Stengers' argument implies that there are at least two different meanings of the term “bias.” When viewed from the perspective of the ideal limit theorem, bias is viewed negatively and unproductively as anything that distorts the comparisons between groups. Thus defined, bias can potentially be eliminated using technical procedures and checklists, but bias can also be defined in terms of a value‐driven perspective on what is worth studying or taking into account. This kind of bias cannot be eliminated. It is unavoidable—and potentially productive and even necessary. Indeed, it could be argued that without bias, there would not be any truths at all. This stance was set out by Thomas Kuhn in his classic text *The Structure of Scientific Revolutions*, in which he showed that at any given time, scientific inquiry is both shaped and constrained by a particular collective framing of a problem and agreed empirical methods for studying it; only when this agreed approach fails to answer newly emerging questions do some scientists break away from the collective narrative and seek to establish a new paradigm.^42^

At first sight, the two views on bias described above appear incommensurable. Stengers' perspectival kind of bias might get her to a particular research question and a particular RCT, but this is a different kind of bias from the biases of the clinicians who allocate the sicker patients to the active drug by peeking at the randomization code or of the researchers who decide not to publish the study after all because it produced negative findings. Yet, in several ways, Stengers' perspective teases out and challenges the assumptions that underpin the truth concept dominant in EBHC.

Firstly, in line with Stengers' critique of an alleged disinterested science, the ideal limit theory dismisses or overlooks a subjective element, such as experience, will, consciousness, agency, or indeed interests. Between the intervention and the outcome, unknown chains of multiple material causes (chemicals, DNA, and heat) and human causes (patient choices and social interaction) are lumped together in pursuit of a single intervention‐outcome correlation. Patients and clinicians are understood and treated to behave like dices, billiard balls, and beings without internal life tending towards ideal limits as the rules out there, which apparently govern things like behaviour, agency, and interests. If RCTs are assumed to be able to find those rules, what reasons are there to believe this? Are human interests just epiphenomenal to a material world^43^?

Secondly, in contrast to Stengers' view, the ideal limit theorem assumes the possibility of full separation between the observer and the human research participant. A position of noninteresse is deemed feasible. It is assumed there are facts out there, with the potential to be discovered by disinterested, objective observers (as long as they work hard enough). Measurement is considered to be attainable without disturbing the topic of enquiry and the ideal limit it tends to. Is this indeed how the world works and if so what proofs or reasons do we have to support this? The bias that should be avoided when comparing groups would not necessarily require a disinterest of the observer, but at least an equal interest in both groups, *equipoise*. Yet even the most ardent EBHC proponents view this as unrealistic as this would dismiss the role of the two other EBM pillars (clinical experience and patient values) and violating what Sackett called “trust in the physician‐patient relationship”^44^ confirming Stengers' point.

Thirdly, Stengers' inter‐esse invokes the idea of goal‐directedness of scientists feeding back and affecting their activities in real time. We suggest clinicians tend not to think in keeping with statistical causation, but more in terms of Aristotle's causes. Dispositions (an innate power of entities to cause)^45^, ^46^ and final causes (purpose defining what things are and can do) are typical in clinical practice.^47^, ^48^ Patients are considered to have tendencies (towards getting better or not) and not to behave like dice (either get better or worse and nothing in between). At the same time, actions are not left to abilities and pure chance. Means are pursued to reach certain ends. Equally, a kind of feeding back is assumed in the ideal limit theorem. There is not just the belief that average outcomes in the long run tend to their ideal limit; the ideal limit is used to make predictions for other patients and events that were not in the research study. This supposes that somehow a future limit has some kind of an impact on individual patients today. It is perhaps remarkable that patients, new treatments, even whole complex interventions already tend towards an ideal limit that will only be revealed in the long run.^17^ But how could and why could this happen?

Fourthly, the ideal limit is considered to be stable over time and pointing in one direction. This is underpinned by the assumption of a deterministic world view with an eventually predictable future if only we knew all properties and conditions of the world. Current methods of measurement may be failing and human interest blocking the view, yet all events in the past, present, and future are fixed already. That reality not only conflicts with current understandings of particle physics that see only probabilities as deterministic, and the occurrence of actual events as fundamentally unpredictable. More importantly, it conflicts with the concept of human free will.^43^ In a fully deterministic world, there is no place for human interests. Is reality really like that? Stengers' notion is one that questions a fixed reality by stressing the importance of interaction and agency.

Fifthly, Stengers draws attention to framing as interests shape concepts and ultimately facts. Defining diseases, interventions, and outcomes (for example, when designing RCTs) is essentially a subjective endeavour. A purification process is needed^49^, ^50^ to describe the specific problem as seen in real patients in one of the many categories as defined by the medical community. The ideal limit comes with the expectation that these in one way or the other correspond to a real world. But why is that acceptable? The ideal limit may help to deny any claims of effect from a certain framing in the long run, but in itself, it does not provide any assurance that framings overlap with those in an underlying reality.

## 5 TOWARDS A PLURALISTIC UNDERSTANDING OF TRUTH IN EBHC

The assumptions underlying the ideal limit theorem are largely metaphysical; they can be neither proven nor refuted empirically. The EBHC community could very well proclaim that the world is deterministic, that our framings reflect how reality is, that an understanding of inner life or mental causality is unnecessary, and that these beliefs are justified as EBHC has been so successful.

However, the ideal limit theorem does not suffice for what EBHC intended to do.^34^ Yes, it provides a truth concept for large groups and frequent events (requiring a belief that similarity can be established unequivocally). But conceptually, it cannot deal with the single case scenario in the clinical encounter—the original situation for which EBHC was developed. In that context, a patient and a clinician need to overcome the so‐called philosophical problem of induction, the inability to predict the future. They will have to make a risky inference; hence, even when the premises are correct, the conclusion and outcome may not be.

As the problem of induction cannot be overcome fully, making inferences is about evasions: finding ways of reasoning to evade the problem and achieve the best prediction possible.^22^ In EBHC, the dominant evasion is the frequency‐type evasion. This evasion assumes we can be right in say 95% of the cases in the long run.

Obviously in the clinical encounter, this kind of reasoning is problematic: there is no frequency of a single event. It is impossible to say whether a single patient is part of the group who will benefit or part of the one that does not. The ideal limit is about the tendencies of a group as a whole, not of individuals in that group. In practice, other evasions are needed^51^ that can embed frequency data in the single case, such as Bayesian (updating beliefs),^22^ mechanistic (how does it work),^52^, ^53^, ^54^ and again means‐ends reasoning (working towards a goal).^47^, ^48^ Evidence‐based health care teachers are keen to educate medical students in Bayesian reasoning because it can be applied to individuals, focusing chiefly on how probabilities of a diagnosis are conditional on certain signs, symptoms, and wider determinants.^55^

It follows that if (and to the extent that) the goal of EBHC is to support reasoning in the clinical encounter, then the ideal limit as the sole concept of truth appears to be both conceptually and empirically insufficient. The cognitive biases observed in highly controlled lab studies as described by Kahneman and Tversky were already contested in the complex reality of clinical practice^33^, ^37^, ^56^ that makes attempts to “debias” clinicians^57^ futile.

As Norman and Eva^37^ write,

“Firstly, thinking about cognitive bias solely as a source of error is inconsistent with the psychological literature on the subject. In psychology, heuristics and biases are viewed as efficient mental strategies with which to deal with an uncertain and ambiguous world. On many occasions they work; occasionally they fail. But they are not intrinsically bad. Secondly, although many (but not all) of these biases have been established experimentally, this occurred in situations in which variables could be manipulated singly and clever manipulations were designed to induce error for the sake of determining if the biases exist. Even experimentally it is hard to observe ‘biases’ in situations where the biases lead to the correct answer. By contrast, when trying to conduct a retrospective examination of a diagnostic error, there is no way to deduce the presence or absence of any error as errors are overlapping and there is no process trace to indicate when and if a particular error arose in the diagnostician's mind.”

This more nuanced and positive perspective on bias resonates with Stengers' notion of “interesse” and raises the question whether there are alternative truth concepts that will help us to differentiate valid inferences from less valid ones while overcoming the challenges mentioned above.

This is critical not just for the clinical encounter. The same is true in other contexts, such as policymaking—perhaps even more so.^58^ Value‐laden, situated activities, dealing with nonfrequent and complex challenges, need more than just the ideal limit theorem. They require “making explicit the premises and values on which each side has built its case.^59^”

Philosophers tend to deal with truth in general and not with single case scenarios. As clinicians, however, we deal with the latter. Accordingly, we would like to illustrate how different philosophical conceptions of truth could come in to play using the following case as an example. The case is fictional yet inspired by three actual clinical encounters that one of the authors (SW) had.

### 5.1 Case

*On a Sunday afternoon at an urgent care centre in a UK hospital, SW sees Ms T, who presents with symptoms of a sore throat. She is 25‐year old and tells SW that it started 2 days before. She may have had a fever, but she is not sure. Drinking is painful. She does not have any other medical conditions, but she does smoke. SW asks what her main concerns are and why she has come in today. She tells him that her housemate got antibiotics from her GP 10 days ago; the GP (allegedly) said that it might be scarlet fever. Ms T's main worry is whether or not she has scarlet fever and whether she needs to start antibiotics. On first impression, the patient does not appear to be very unwell. She is not drooling or coughing, and her breathing is normal. SW sees on the computer system that the triage nurse took her temperature. It is 37.8. She is able to open her mouth on examination, and SW sees two enlarged tonsils, with some small white spots. They appear quite symmetrical. Her skin looks a bit red in her neck, but the lighting is not optimal so it is hard to be certain.*

At this point SW is not quite sure how to proceed regarding her question about scarlet fever. SW has trained and worked as a GP in the Netherlands and is aware of the guidelines both there and in the UK. Based on what he remembers from them, he understands that scarlet fever^60^ is a notifiable condition in the UK for which antibiotics should be considered and swabs taken while in the Dutch guideline notification, swabs or antibiotics are not necessary routinely.^61^

*He says he would like to discuss her case with a colleague in the next room. E is OK with that and he goes to the next room where he sees AC, another GP. SW explains the case to her briefly. She says she is not sure either, but as Ms T appears otherwise well, she thinks scarlet fever is not on top of the differential and she would await events. SW agrees with this and returns to Ms T. He explains that he and his colleague think that a viral infection is more likely than scarlet fever now and that she can await events without antibiotics as it is likely that the symptoms will settle over the next week. He advises her to see her GP if things do not improve or come back to the urgent care if she feels more unwell or cannot swallow anymore.*

### 5.2 Analysis

To inform the decision whether or not to prescribe antibiotics, SW could look at the effectiveness of antibiotics from a Cochrane systematic review of RCTs^62^ as reported in the Dutch guideline. This shows that after 7 days of antibiotics use, the symptoms had settled in 87% of the patients, compared with 82% in the placebo group. That finding is however of little help to Ms T as she cannot have the antibiotics 100 times in parallel (in which case she should recover about 87 times if she would take antibiotics and 13 times not). She will only have antibiotics once, and there is no way to tell beforehand whether she will be part of the 87% who improve on antibiotics or the 13% who do not (similarly, 82% who recover or 18% who do not if she did not take them). Thus, the ideal limit theorem does not function in the single case scenario as a way of predicting the future.

However, SW can use the frequentist findings from the Cochrane review to update his a priori belief about what is likely to happen to his patient (hence, taking a Bayesian perspective). Yet, in itself, this is still not good enough to draw a conclusion, as there are many other eventualities to consider—such as the likelihood of Ms T developing suppurative or nonsuppurative complications, allergic reactions, and bacterial resistance. Some of those can be updated with frequentist data of course, but they would leave SW with many conflicting and disjointed inferences. To make a decision, he needs to incorporate concepts of truth that can deal with the practical problem facing him and his patients in the here and now.

SW tries to make a kind of inference that is limited by what he is trying to achieve for the near future, namely, Ms T, achieving relief of her symptoms or preventing complications. Here, we see a kind of goal directness or mean‐ends reasoning^47^ delimiting what SW can infer from all possible inferences. Aristotle referred to these as final causes,^63^ purposes pushing back on reality. He also proposed the concept of orexis, in the words of Nussbaum an inclination, desire, reaching out to explain that actions are guided by both goal‐directness and the “limits imposed by the world of nature.”^64^ What's more, SW's goal‐directedness is a certain kind of goal‐directness: it is virtuous, the aim is to do good, instead of bad. This is reflected in work by several authors^10^, ^12^, ^65^ who question whether the application of epistemic virtue (or vices), morality, justice, and human rights differentiate truth from untruths.

What SW can know and infer is further limited by his reasoning, he has no certainty of anything “out there.” This points to a kind of correspondence concept of truth: (transcendental) idealism as argued by Kant,^66^ Lonergan,^67^ and many others. They point out that to make a good inference, SW is dependent on reasoning. Yet many less explicit kinds of knowing, such as tacit knowledge and practical knowledge (as captured in mindlines^68^, ^69^), and less rational kinds of thinking (intuitively, fast,^70^ subconsciously, or using heuristics^71^, ^72^ and gut feelings^73^) do not fit coherence, idealism, or any other dominant theories of truth very well. Rather, they are situated, individual, and oriented to answering the moral question “what is the best thing to do for this patient at this time, given these contingencies along with wider evidence?”

In consulting his colleague, SW uses consensus (albeit on a very small scale) as a concept of truth to limit all the possible inferences. In this view, it is not just that two GPs know more than one, their agreement actually makes things more true. This is somewhat reflected in the Bolam test, a criterion in English medical law: what a community of clinicians considers a reasonably competent clinician would infer in a specific case is considered true.^74^ But consensus between patients and clinicians is often not clearly expressed. A polyphonic concept of truth as developed by Bakhtin, where the combined utterances of participants in a meaningful conversation limit the possible inferences, is perhaps better suited. SW is never an objective observer and cannot be disinterested as Stengers points out in the more general case. Throughout the interaction between SW and Ms T, the concern about scarlet fever emerges, not out of agreement, but through interaction it becomes an entity that needs to be addressed.

Pragmatism, the idea that what works or what is useful is true as developed by William James^75^ and others, might fare better in the single case scenario. However, it is not so clear for SW to see what “works.” Following frequentist data, giving or not giving antibiotics both “work” in reducing symptoms in 7 days. Equally, to the complication rate is so low (the number needed to treat for rheumatic fever is about 1 in 1,4 million^76^) that it is hard to see how diagnosing or not diagnosing scarlet fever (with the result of notifying or not notifying) in Ms T case achieves anything for her or is useful for anyone else. Philosophers have drawn attention to the point that in practice many actions “work,” and it might be more important to focus on what does not work or has negative consequences (i.e., negative pragmatism^77^).

In this case, SW tries to adhere broadly to the relevant guideline (as he remembers it) to make the best inference for Ms T but becomes puzzled as guidelines from two countries conflict. Both of them are based on about the same international systematic reviews and RCTs but through the guideline development process somehow ended up with very different recommendations. A constructivist concept of truth operates here. The Dutch guideline makers chose to put,^61^ for instance, less emphasis on studies from before 1960 since then rheumatic fever (and other nonsuppurative complications) has become so rare, that a GP will most likely never see it. Moreover, SW has to take into account that a UK trained colleague seeing Ms T the next day has no awareness of the Dutch guidance. As a result, SW needs to align any inference on scarlet fever with UK practices. Furthermore, as he deviates from what Ms T might find online on scarlet fever, SW can explain more carefully his reasoning, bringing in elements of multiple concepts of truth. For instance, that he is not convinced by the small risk of serious complications or the added effect of antibiotics in the long run (kind of ideal limit via a Bayesian evasion), that his colleague broadly agrees with him (an element of consensus), that he sees diagnosing scarlet fever as not useful in helping the patient achieve her purpose (kind of goal‐directed pragmatism) and has noticed other recommendations based on similar evidence (a kind of constructivism).

## 6 FINAL REMARKS

In this paper, we have argued that bias is in a dual, complex, necessary, and unproductive as well as a productive conjunction with truth. We contend that a shift is needed in EBHC from a narrow focus on bias based on the ideal limit concept of truth towards more extended understanding of many theories of truth based on different philosophical positions.

We realize that this kind of thinking about other truth concepts is still somewhat preliminary and more work is needed to develop a more detailed comprehension of bias and truth in EBHC. But we would argue that evidence‐based practice could gain much by starting to debate, teach, and extend concepts of truth to develop a better understanding of proper inference.

It appears promising that decision theories that are based on a less deterministic view on the world seem to be able to model human decision‐making, *including* the biases found by Kahneman and Tversky such as the conjunction fallacy (specific conditions are considered more probable than general ones).^43^, ^78^ On the basis of these theories and assumptions, a mathematical psychology model was proposed that deciding is not about picking from a range of fully rationalized preferences, but more like making one of many vaguely defined, dynamic, interrelated thoughts superposition stable (real) by a kind of goal directness.^36^

Categorizing and analysing bias is still necessary to aid research that is guided by the ideal limit concept. But to advance evidence‐based practice, and provide arguments in a post‐truth world, we would argue that an exploration of multiple truth concepts is needed too. It would help to provide underlying theory to qualify the perceived bias, to understand how the ideal limit concept works in reality, and to further study how the different truth concepts interfere with each other.^74^

Furthermore, as explicit knowledge (of varying quality) is more widely available through the internet, the role of the clinician as provider of knowledge changes. How to differentiate truths from untruths and how to make a right inference for a particular case will become more important. Future clinicians should be educated and appropriately skilled to discuss underlying theories and assumptions of truth and bias in order to reconcile the ideal limit with human interests in the clinical encounter.
